# An After-School Football Session Transiently Improves Cognitive Function in Children

**DOI:** 10.3390/ijerph20010164

**Published:** 2022-12-22

**Authors:** Daniele Magistro, Simon B. Cooper, Ruth Boat, Fabio Carlevaro, Francesca Magno, Cristian Castagno, Martina Simon, Giovanni Musella

**Affiliations:** 1 Department of Sport Science, School of Science and Technology, Nottingham Trent University, Nottingham NG11 8NS, UK; 2 Polo Universitario Asti Studi Superiori (Uni-Astiss), 14100 Asti, Italy; 3 Dipartimento di Scienze della Vita e Biologia dei Sistemi, University of Torino, 10124 Torino, Italy

**Keywords:** cognition, children, physical activity, football, attention, memory

## Abstract

The aim of the present study was to evaluate the effect of a real-world after-school football session on subsequent cognitive function in primary school children. Following ethical approval, 100 children (aged 8–9 year) from the same after-school football club were randomly assigned to either an intervention (60 min football activity) or control (continued to rest) group. Cognitive function (selective visual attention, short term memory and long-term memory) was assessed prior to, immediately following and 45 min following the football session (and at the respective timepoints in the control group). Data were analysed via two-way (group * time) mixed methods ANOVA. The pattern of change in all domains of cognition over time, was different between the football and control groups (group * time, all *p* < 0.001). Specifically, performance on all cognitive tasks was greater immediately following the football session in the intervention group compared to the control group (selective visual attention, *p* = 0.003; short-term memory, *p* = 0.004; long-term memory, *p* < 0.001). However, there was no difference between the group 45 min following the football session (*p* = 0.132–0.393). These findings suggest that an after-school football session enhances cognition immediately post-activity in primary school children.

## 1. Introduction

Cognitive function can be defined as a wide range of brain mediated functions and processes, and is often categorised in to six main domains: memory, attention, perception, executive function, psychomotor skills and language [1]. Given this definition, it is therefore unsurprising that much interest has been generated in the education sector and the academic community as to the factors that influence cognitive function in young people. In particular, given the proposed links between enhanced cognitive function and the subsequent beneficial effects of learning and academic achievement [2,3,4,5,6], strategies to enhance cognitive function are of particular importance in children and adolescents.

A number of potential influences on cognitive function in school aged children have been examined, such as nutrition (most commonly breakfast consumption) [7,8] and physical activity [9,10]. Physical activity is of particular interest, not least because globally 50% young people do not meet the recommended levels of 60 minutes of moderate-vigorous intensity physical activity per day [11]; but also due to the multi-faceted health benefits that can be gained from achieving physical activity guidelines [12,13]. It is also important to consider and evaluate physical activity models that are ecologically valid, i.e., those that young people actually participate in. Data suggest that the activity patterns of young people are high intensity and intermittent in nature [14], even when taking part in a structured physical activity intervention such as The Daily Mile [15]. These high intensity intermittent activity patterns are similar to those seen in team games such as football [16]. Furthermore, team games are a form of physical activity that young people enjoy, which is vital for long-term adherence [17] and ultimately for young people to gain maximal benefits from participation in physical activity.

An extensive body of literature suggests that an acute bout of physical activity enhances subsequent cognitive function in young people [18,19,20]. However, due to the additional control over experimental conditions that is offered, many of these studies utilise activity protocols that are administered, and closely monitored, by researchers. Therefore, it can be argued that these protocols lack ecological validity, and that the effects of physical activity, when conducted in the real-world setting, on subsequent cognitive function warrant further investigation. Furthermore, a number of factors have been suggested to influence the acute effects of physical activity on subsequent cognitive performance, such as characteristics of the physical activity (e.g., modality, intensity and duration), the fitness level of the participants, and the timing of cognitive measurements following physical activity [19]. It is likely that these key variables will differ in real-world settings (compared to researcher-controlled settings, as used in previous studies); therefore exploration of the effects of real-world physical activity on subsequent cognition in young people is required.

Some researchers have examined the acute effects of team games-based physical activity on subsequent cognition in young people [21,22,23]. However, the findings of these studies are mixed, whereby beneficial acute effects of 60 min basketball on working memory and executive function [21], and 20 min football on attention and executive function [22], have been demonstrated; whereas other work has demonstrated no acute effects of a 60 min football session across a range of domains of cognition [23]. It is likely that such discrepancies are the result of variations in the intensity and duration of the physical activity sessions between studies. It is also important to note that the physical activity sessions were researcher-led and monitored in each case, thus potentially lacking ecological validity. Furthermore, the studies which have examined games-based activities [21,22,23] were all conducted in 11–13 year olds. Childhood and adolescence represent periods of rapid growth and changes in young people [24,25], including of the brain [26]. Thus, it is unsurprising that the acute effects of physical activity on subsequent cognition have been suggested to be different between younger children and adolescents [19,27]. Furthermore, the domain of cognition assessed is an important consideration. Consideration must be given to the importance of the cognitive domain within everyday life, and the underpinning theory as to why a certain cognitive domain may be affected by physical activity. For example, attention is a key element in the identification of stimuli, and in football players visual attention is fundamental to understand the context and environment for decision making [28]. Furthermore, complex motor tasks, such as team sport activities, stimulate the activation of the cerebellum, affecting speed and accuracy of attention and memory tasks. Thus, it is important that future work addresses the dearth of evidence regarding the effects of (real-world) team games activities on the important cognitive domains of attention and memory in children.

Therefore, the aim of the present study was to examine the effects of an acute bout of football activity, administered in real-world conditions, on subsequent cognitive function (assessing the important domains of attention and memory) in children. Based on the literature to date, it was hypothesised that an acute bout of football activity would enhance subsequent attention and memory in children. 

## 2. Materials and Methods

### 2.1. Study Design

Following ethical approval from the host institution (University of Torino, ID 100949), a between subjects experimental research design was utilized in this study. Children who regularly participated in an after-school football club were divided in to intervention and control groups. The intervention group took part in their usual 60 min after-school football training session, whilst the control group rested. The control group did not take part in the football session and did no physical activity during this time; however, social interaction between the children in control group was allowed (i.e., passive social activity). Informed consent was obtained from children’s parents/guardians, and assent was provided by the children themselves.

### 2.2. Participants

Power analysis (G-Power software) [29] showed that at least 36 participants for each group was sufficient to gather 80% power with a *p* value of 0.05 on our measures. Consequently, 100 boys aged 8–9 years volunteered to participate in the study. All children were recruited from the same after-school football club in the north of Italy and were randomly assigned to the intervention (football) group (*n* = 50) or to the control group (*n* = 50). There were no differences between the groups in terms of the number of after-school football sessions attended per week (football group: 2.3 ± 0.7, control group: 2.3 ± 0.6 sessions per week, *p* = 0.527), or the number of hours spent in football training per week (football group: 3.9 ± 1.2, control group: 3.8 ± 1.1 hours per week, *p* = 0.550). 

### 2.3. Experimental Protocol

A battery of cognitive function tests was completed at three different time points: before the training (baseline), immediately following the football session, and 45 min following the football session; and at the respective time points in the control group. Following the baseline tests, the intervention group completed their usual 60 min after-school football session, whereas the control group continued to rest. 

### 2.4. Football Session

The intervention consisted of 60 min of football training, delivered via the usual after-school football activity that participants regularly participated in. The training consisted of a warm-up activity (10 min), followed by three game activities:

Activity 1 (15 min): a square with dimensions of 25 m × 25 m was created and two teams “a” and “b” (5 vs. 5) were created. Team “a” started on the perimeter of the square, while team “b” started inside the square. Team “b” had as its objective the maintenance of possession of the ball, through passes between teammates. The coach nominated one child at a time from team “a” who had to enter the field to steal the ball from the opposing team “b”. If within 7 seconds the child was unable to do so, the instructor would call a second child from team “a” who had the goal of helping the first teammate. Each time the ball was successfully brought outside the square by team “b” the teams roles were reversed, and the game restarted. 

Activity 2 (15 min): in the second game, after having prepared three 10 m × 10 m small pitches and a goal in front of it (15 m from the square pitch), four children were placed on the side of each square and one child inside each square. The goal of the game for the children placed on the perimeter of the square was to keep possession of the ball and, once they reached 5 passes, the child placed on the side closest to the goal could go to score via a 1 vs. 1 against the child inside the square. The goal for the child inside the square was to steal the ball from those outside and, if successful, they would change position with the child who lost possession of the ball. 

Activity 3 (20 min): In the third activity, a pitch of 25 m × 20 m was set up with a goal (2 m high by 3 m wide) on one side of 20 m and two small goals on the opposite side. Two teams (“a” and “b”, each consisting of 5 children) were created and a “joker” was chosen. At the start of the game, two players plus a goalkeeper from each team entered the playing area. The aim of the game was to score in the opponent’s goal. The “joker” played for the team in possession of the ball at all times, and had to touch the ball prior to the team being allowed to score a goal. When a goal was scored, the children rotated positions and the “joker” was changed. 

### 2.5. Cognitive Function Tests

The battery of cognitive function tests consisted of a test of selective visual attention, and Rey Word Recognition Test, to assess both short-term and long-term memory. Participants had the tests fully explained to them, and they were familiarised with the tests during the first experimental visit. The tests were completed prior to the football sessions, and immediately and 45 min following the football, sessions (and at the respective times in the control group); with parallel versions of the tests used for the repeated measurements. The battery of tests took approximately 10 min to complete, and was completed in the following order:

Selective Visual Attention: The Selective Visual Attention test [30] assesses this fundamental cognitive function that describes the tendency of visual processing to be confined largely to stimuli that are relevant to behaviour [31]. In the test participants were asked to identify the 12 correct geometric symbols (which matched a target symbol) from a sheet with 80 symbols, in a maximum time of 60 s. The target (correct) symbol was a rhombus, showed at the top of the sheet. The participant received one point for every correct answer, resulting in a score from 0 to 12.

Rey Word Recognition Test: This is a test of verbal memory, and can be used to assess both immediate and deferred recall [32]. A list of 15 words was read aloud to participants at a rate of approximately one word per second. Immediately following the presentation of the words, participants were handed a sheet containing the 15 targets and 15 distractor words, and asked to circle the words that had been read to them (short-term memory). Fifteen minutes after the immediate recall trial, participants were again asked to recall as many words on the list, of 15 targets, as possible (long-term memory). The criterion score was the number of target words correctly recalled.

### 2.6. Statistical Analysis

Data were analysed using SPSS (IBM SPSS Statistics, Version 28, Armonk, NY). To assess changes in cognitive function over time between the intervention and control groups, a two-way (group [football vs. control] * time [pre vs. immediately post vs. 45 min post]) mixed methods ANOVA was used, with repeated measures for time. Where a significant group * time interaction was found, follow-up independent samples *t*-tests (Bonferroni corrected) were used to compare differences between the intervention and control group at each time point, with effect sizes for between group differences calculated as Cohen’s *d*. Data are reported as mean ± standard deviation (SD) and statistical significance was accepted as *p* < 0.05.

## 3. Results

Data for each of the cognitive function tests, in the intervention and control groups at each time point, are displayed in Table 1.

### 3.1. Selective Visual Attention

**Figure 1:**
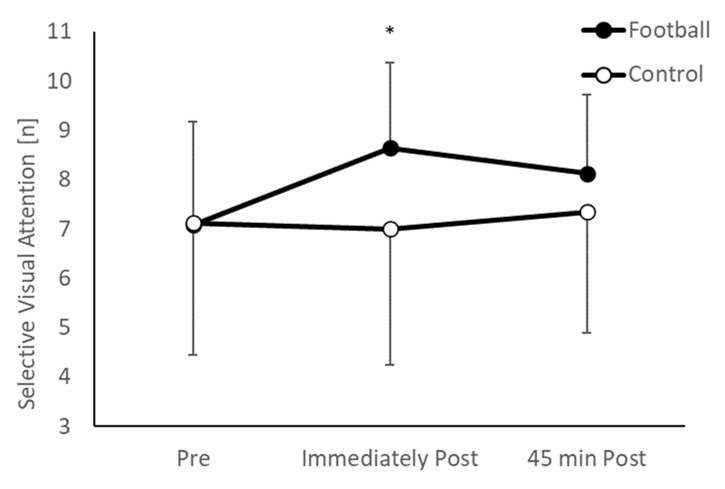
Selective visual attention ([n] = number of correctly identified symbols) in the football and control groups across the morning (group * time interaction, *p* < 0.001; * football > control, *p* = 0.003).

Overall, there was no difference in selective visual attention between the intervention and control groups (main effect of group, *p* = 0.097). However, the pattern of change over time between the groups was different (group * time interaction, *F*_(2,196)_ = 8.119, *p* < 0.001; Figure 1). Specifically, whilst there was no difference between the groups pre-exercise (*p* = 0.941, *d* = 0.01), the football group displayed significantly greater short term memory immediately following the football session (*t*_(98)_ = 3.014, *p* = 0.003, *d* = 0.60), but this effect was not evident 45 min following the football session (*p* = 0.132, *d* = 0.30).

### 3.2. Short Term Memory

**Figure 2:**
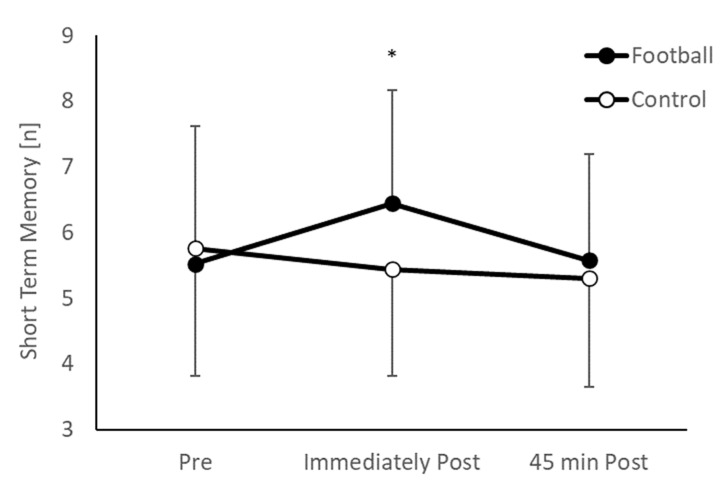
Short term memory performance ([n] = number of correctly recalled words) in the football and control groups across the morning (group * time interaction, *p* < 0.001; * football > control, *p* = 0.004).

Overall, there was no difference in short term memory between the intervention and control groups (main effect of group, *p* = 0.283). However, the pattern of change over time between the groups was different (group * time interaction, *F*_(2,196)_ = 10.757, *p* < 0.001; Figure 2). Specifically, whilst there was no difference between the groups pre-exercise (*p* = 0.554, *d* = −0.12), the football group displayed significantly greater short term memory immediately following the football session (*t*_(98)_ = 2.987, *p* = 0.004, *d* = 0.60), but this effect was not evident 45 min following the football session (*p* = 0.393, *d* = 0.17).

### 3.3. Long Term Memory

**Figure 3:**
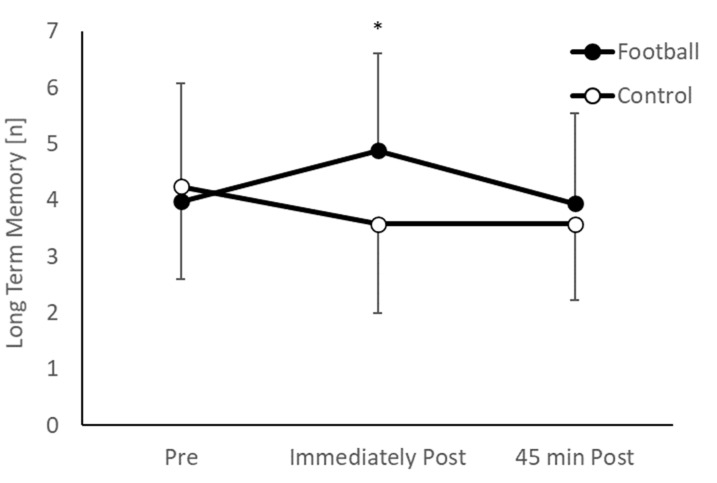
Long term memory performance ([n] = number of correctly recalled words) in the football and control groups across the morning (group * time interaction, *p* < 0.001; * football > control, *p* < 0.001).

Overall, there was no difference in long term memory between the intervention and control groups (main effect of group, *p* = 0.071). However, the pattern of change over time between the groups was different (group * time interaction, *F*_(2,196)_ = 11.312, *p* < 0.001; Figure 3). Specifically, whilst there was no difference between the groups pre-exercise (*p* = 0.465, *d* = 0.15), the football group displayed significantly greater short term memory immediately following the football session (*t*_(98)_ = 3.949, *p* < 0.001, *d* = 0.79), but this effect was not evident 45 min following the football session (*p* = 0.181, *d* = 0.27).

## 4. Discussion

The main findings of the present study were that an acute 60 min bout of football, administered via an after-school football club, enhanced subsequent cognition in primary school children aged 8–9 years old. Specifically, children in the football group displayed enhanced selective visual attention and verbal memory (both short-term and long-term) immediately following the football session, when compared to the control group. A further key finding of the present study was that whilst the football session enhanced cognitive function when assessed immediately post-exercise, the beneficial effects were not evident when cognition was assessed 45 min following the football activity.

A key finding of the present study is that an ecologically valid, real-world, after-school football session enhanced subsequent cognitive function in primary school children. This is important given that many previous studies in this area have used physical activity protocols that are closely controlled and monitored by the researchers, and thus potentially lack real-world applicability. When the physical activity is influenced by the researchers, it is likely that the intensity of activity and the nature of the activity itself will be different compared to real-world settings. This is a very important consideration given the potential for these variables (such as the intensity and modality of activity) to moderate the subsequent effects of physical activity on cognition in young people [19]. Therefore, the present study provides important novel evidence that a real-world physical activity intervention enhanced subsequent cognition in children.

The present study demonstrates enhanced visual attention and memory performance in children following participation in the football session, when compared to the control group. Team games such as football involve important top–down cognitive control and the ability to direct movements and automatic behaviours [33]. These activities stimulate cognitive elements related to visual-spatial attention (such as visual search). Furthermore, football skills involve complex cognitive abilities to anticipate contextual activities such as team-mates and opponents’ behaviours, and strategies to adapt to changing task demands [34,35]. Thus, the football activity may have induced these improvements in cognition due to the cognitive processes involved in the activity itself. Furthermore, these cognitive domains have implications for both sporting performance and learning/educational outcomes in schools. In terms of sporting performance, any sport that requires decision making (such as team games) relies on cognitive function for successful performance [19,36,37,38]; and in particular the domains of visual attention (detecting task relevant cues from the environment, such as movement of the ball and teammates/opponents in football) and memory (recalling tactical information) as assessed in the present study. Therefore, the enhancements seen immediately following football activity are of interest to youth sports coaches who might wish to, for example, ensure that a warm-up is completed immediately prior to participation in a match, to maximise the cognitive benefits that may be gained. In terms of educational outcomes, these findings also have implications for school policy makers, who may wish to ensure opportunities for physical activity are provided throughout the school day, in order to ensure optimal cognition for learning; ultimately enhancing learning and educational outcomes [2,4]. Therefore, the improvements in visual attention and memory in the present study are of potential interest to both those involved in youth sport, and education.

A further key finding of the present study was that, despite improvements in cognition immediately following the football activity, these beneficial effects were not evident when cognition was assessed 45 min following the football session. The immediate benefits to cognition are in line with the body of evidence suggesting that an acute bout of activity enhances cognition in young people [18,19,20]. However, the lack of an effect 45 min following the football session is in contrast to previous evidence showing that cognition is enhanced 45–60 min following physical activity in young people [10,21,39]. Possible reasons for the discrepancy in findings could be that the participants in the previous studies examining high intensity intermittent [39] and team games [21] activity were adolescents aged 11–13 years, compared to the younger children aged 8–9 years in the present study. Indeed, previous reviews have suggested that age may moderate the acute effects of physical activity on cognition [19]; with the present study tentatively suggesting that the acute effects of physical activity on cognition are more transient in children than adolescents. This area however warrants further research, given the implications of the time course of the cognitive effects for those interested in enhancing cognition and, for example, learning and educational outcomes in young people.

Whilst the present study has a number of strengths, such as the real-world nature of the football session, it is not without limitation. One limitation of the present study is the between-group design, meaning that potential differences between the groups and confounding variables could influence study outcomes. To mitigate this as a limitation, it should be noted that all participants (in both the football and control groups) were from the same after-school football group and demonstrated similar levels of typical engagement with the after-school football club. However, it would be advantageous for future work to adopt a repeated measures, within-group, design, with all participants completing both the football and control trials. Furthermore, the cognitive tests administered in the present study were paper and pencil tests; thus, potentially lacking the sensitivity of more sophisticated computer-based cognitive tasks. However, the administration of paper and pencil cognitive tasks did facilitate the relatively large number of participants (*n* = 100 in total) to be tested simultaneously. Similarly, the use of cognitive tests in this manner is required to examine the acute effects on activity; future research could also examine the effects of longer-term implementation of physical activity interventions on academic achievement, an effect likely mediated through acute enhancements to cognition. Finally, it should be noted that all participants in the present study were boys, and thus the applicability of the findings to girls remains unknown and warrants further investigation. 

## 5. Conclusions

In conclusion, the present study demonstrates that a real-world after-school 60 min football session led to immediate improvements in cognition (across the important domains of visual attention and memory); although these effects were not evident when cognition was assessed 45 min following the football session, when compared to the control group. These findings add to the literature by examining the effects of games-based activity, which is an enjoyable form of physical activity for young people and thus an attractive intervention target, in primary school aged children. These findings have implications for those interested in enhancing cognitive function in young people, such as schools and school policy makers who wish to enhance learning and educational outcomes; in addition to sports coaches who are interested in enhancing sporting performance.

## Figures and Tables

**Table 1:** Cognitive function across the morning in the intervention and control groups. Data are mean.

Test Variable	Intervention Group			Control Group		
	Pre	Immediately Post	45 Min Post	Pre	Immediately Post	45 Min Post
Selective visual attention	7.08 ± 2.69	8.64 ± 2.69	8.12 ± 2.68	7.12 ± 2.66	7.00 ± 2.75	7.34 ± 2.45
Short term memory	5.52 ± 2.09	6.44 ± 1.73	5.58 ± 1.60	5.76 ± 1.94	5.44 ± 1.62	5.30 ± 1.66
Long term memory	3.98 ± 1.89	4.88 ± 1.70	3.94 ± 1.32	4.24 ± 1.65	3.58 ± 1.59	3.58 ± 1.36

## Data Availability

Data are available upon request.
